# Supplementary figures and images for: Comparison the effects of carotid endarterectomy with carotid artery stenting for contralateral carotid occlusion
Source: PLoS One. 2021 May 20;16(5):e0250580. doi: 10.1371/journal.pone.0250580 (PMC8136671; doi:10.1371/journal.pone.0250580)

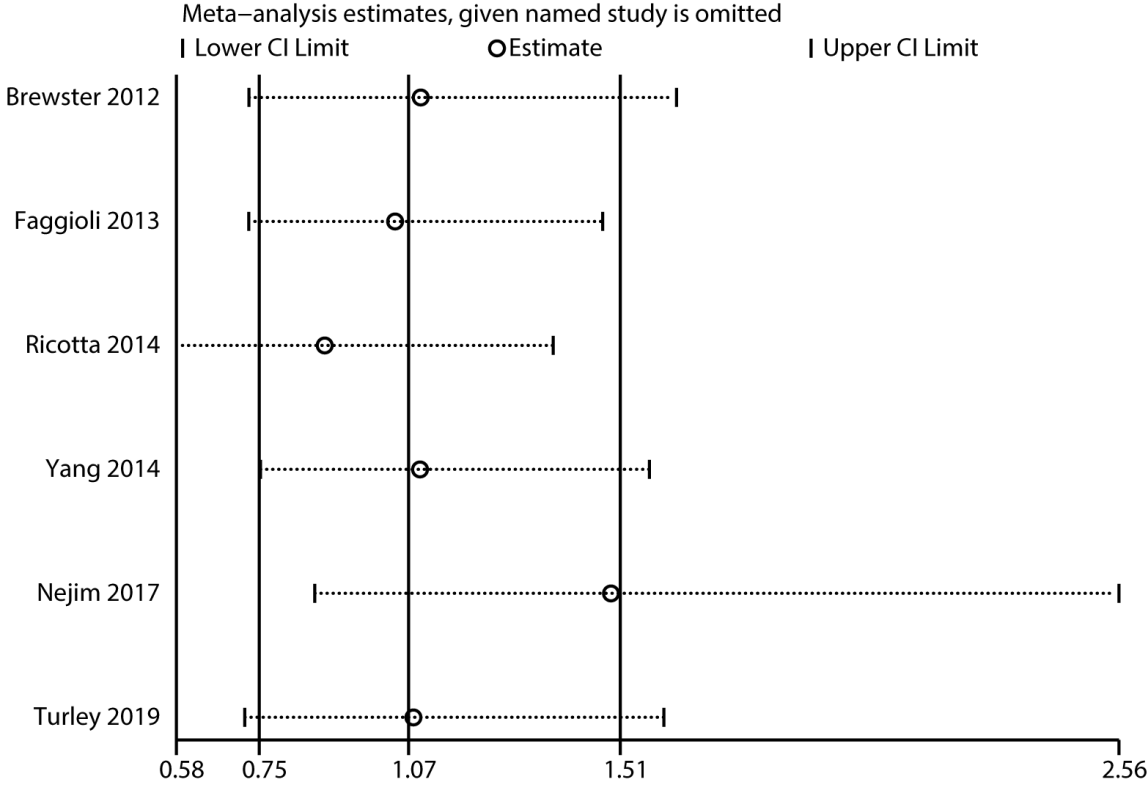


S1 Fig. Sensitivity analysis for stroke

Supplement: S1 Fig — (DOCX) [file pone.0250580.s001.docx]

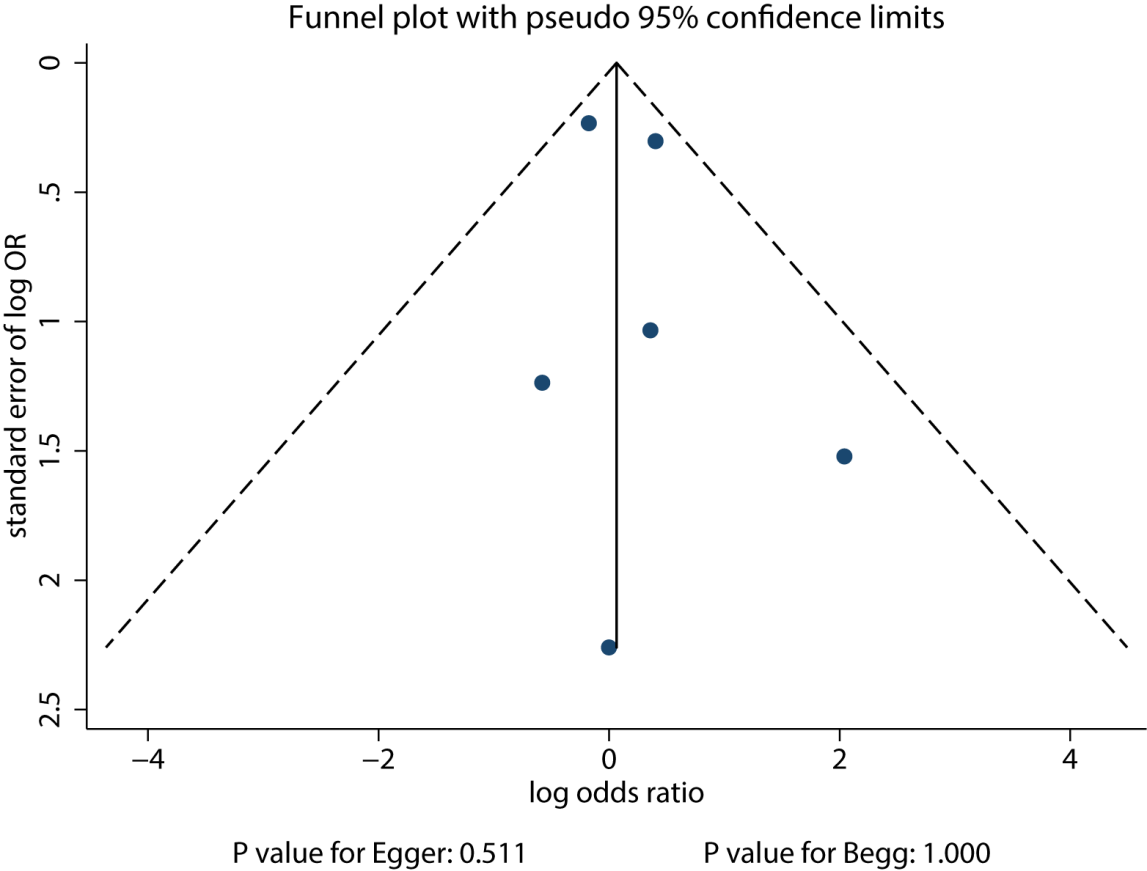


S2 Fig. Funnel plot for stroke

Supplement: S2 Fig — (DOCX) [file pone.0250580.s002.docx]

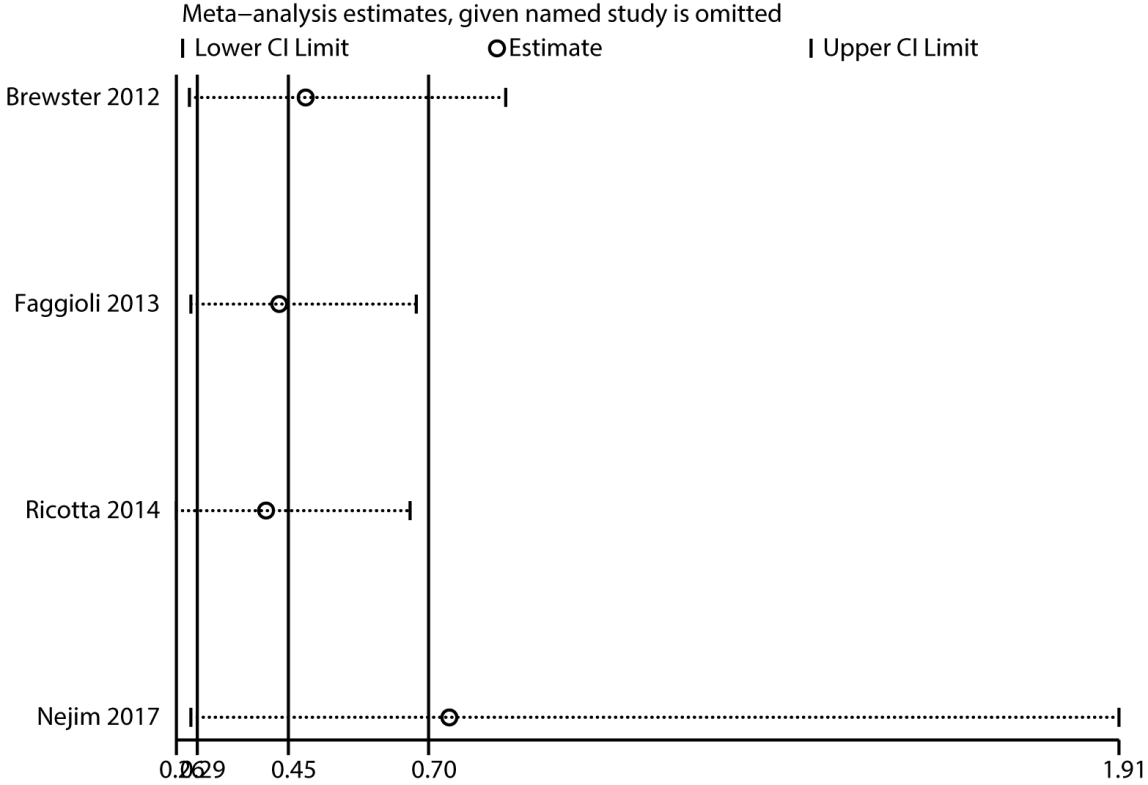


S3 Fig. Sensitivity analysis for death

Supplement: S3 Fig — (DOCX) [file pone.0250580.s003.docx]

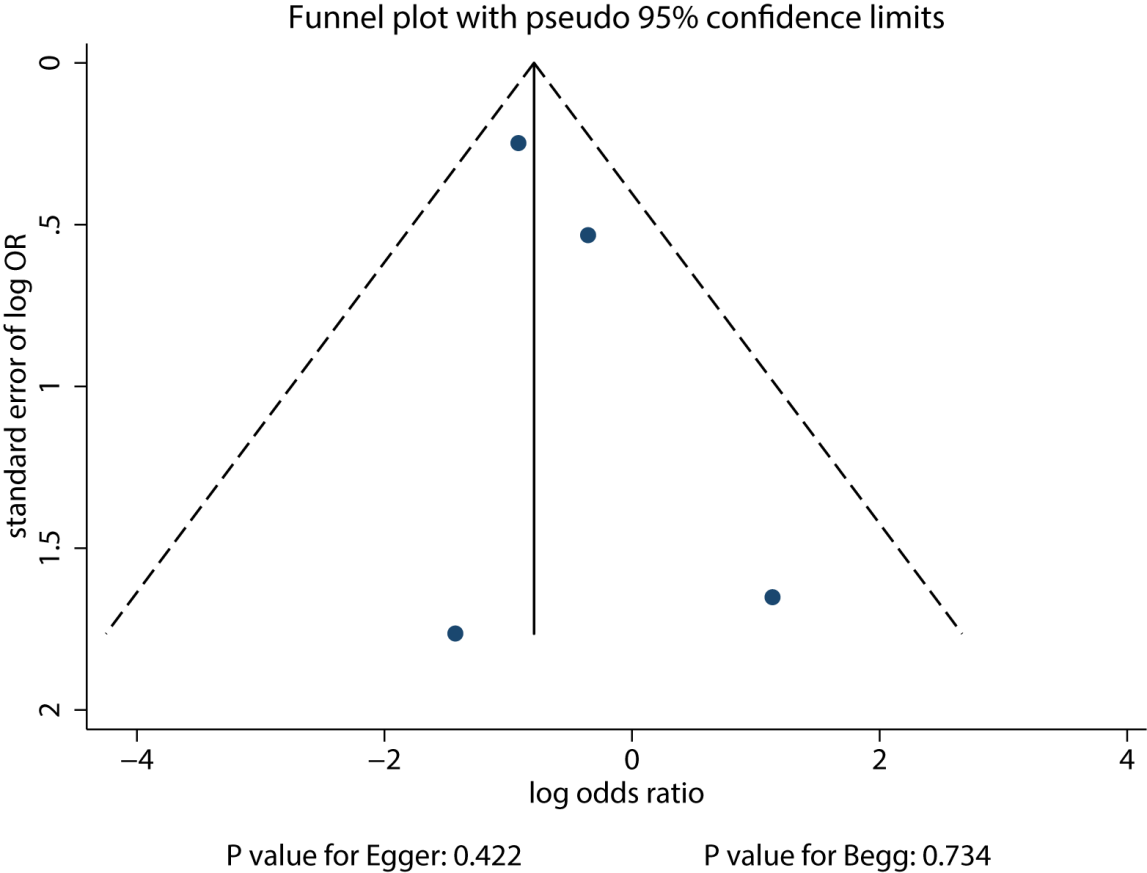


S4 Fig. Funnel plot for death

Supplement: S4 Fig — (DOCX) [file pone.0250580.s004.docx]
