# Supplementary material for: Comparison the effects of carotid endarterectomy with carotid artery stenting for contralateral carotid occlusion
Source: PLoS One. 2021 May 20;16(5):e0250580. doi: 10.1371/journal.pone.0250580 (PMC8136671; doi:10.1371/journal.pone.0250580)
Supplement: Attachment — Submitted filename: Response to reviewer.doc [file pone.0250580.s006.doc]

**Dear editor,**

Thanks for your kind reply, and thanks for the reviewers’ beneficial comments. We have revised the manuscript according to the reviewers’ comments and the point to point response is enclosed to this file. The reply was noted with tracked.

**Response to editor**

Question 1: Please add a table with the assessment of the quality of the included studies.

Response: Thanks for this suggestion, and the quality scores of included studies using Newcastle-Ottawa Scale have already presented in Table 2.

Question 2: Please ensure that your manuscript meets PLOS ONE's style requirements, including those for file naming. The PLOS ONE style templates can be found at

https://journals.plos.org/plosone/s/file?id=wjVg/PLOSOne_formatting_sample_main_body.pdf and

https://journals.plos.org/plosone/s/file?id=ba62/PLOSOne_formatting_sample_title_authors_affiliations.pdf

Response: Thanks for this suggestion, and the manuscript have already updated according to PLOS ONE's style requirements. All of changes in the revised manuscript with tracked.

Question 3: Please review your reference list to ensure that it is complete and correct. If you have cited papers that have been retracted, please include the rationale for doing so in the manuscript text, or remove these references and replace them with relevant current references. Any changes to the reference list should be mentioned in the rebuttal letter that accompanies your revised manuscript. If you need to cite a retracted article, indicate the article’s retracted status in the References list and also include a citation and full reference for the retraction notice.

Response: Thanks for this suggestion, and the reference lists have already updated according to the PLOS ONE's requirements.

Question 4: Please include captions for *all* your Supporting Information files at the end of your manuscript, and update any in-text citations to match accordingly. Please see our Supporting Information guidelines for more information: <http://journals.plos.org/plosone/s/supporting-information>

Response: Thanks for this suggestion, and the captions for supporting information have already added at the end of the manuscript. Moreover, the in-text citations have already updated with tracked.

**Response to reviewer #1**

General comments: Nice written work on very important and contested problem. I wish they show the criteria of patients selected for CEA vs CAS in the studies. I would also like to know why CEA associated with lower mortality. Could it be that patients declined for CEA because of higher surgical risk go for CAS and ending up having more mortality ? Can the authors comment on that if possible.

Response: We appreciate the reviewer given this constructive comments. We have already added the characteristics between CEA and CAS groups in Table 1 if they associated with statistically significant. Moreover, the results regarding the high risk of death in CAS group have already discussed in Discussion section.

**Response to reviewer #2**

General comments: Overall well written article by Sun et al. comparing the effects of carotid endarterectomy with carotid artery stenting for contralateral carotid occlusion in a total of 6953 patients. The study found that CEA and CAS had similar outcomes for patients with CEA versus carotid stenting, while the risk of death was reduced in patients treated with CEA. Overall, these conclusions are similar to a previous meta analysis on the topic by Texakalidis et al. in 2018 involving 6346 patients. The authors report there sample size is larger after addition of an addition retrospective study. However, I do not feel this study adds much to the scientific literature given the additional studies including in this analysis were all retrospective in nature and not RCTs. There is only a difference of approximately 600 patients in the sample size between these two analyses and as such, the similar findings are not surprising.

Response: Thanks for this suggestion. We appreciate the reviewer given this constructive comments. We acknowledge the results of this study were consistent with the study conducted by Texakalidis et al. However, the current study also assessed whether the treatment effects of CEA versus CAS are differing based on age, male gender, disease status, coronary artery disease, hypertension, diabetes mellitus, and smoking. We have already addressed this question in Discussion section.
